# A Study of Cardiogenic Stroke Risk in Non-valvular Atrial Fibrillation Patients

**DOI:** 10.3389/fcvm.2020.604795

**Published:** 2020-11-05

**Authors:** Ziliang Song, Kai Xu, Xiaofeng Hu, Weifeng Jiang, Shaohui Wu, Mu Qin, Xu Liu

**Affiliations:** Shanghai Chest Hospital, Shanghai Jiaotong University, Shanghai, China

**Keywords:** atrial fibrillation, cardiogenic stroke, left atrial appendage thrombus, LA-SEC, risk model

## Abstract

**Objectives:** We attempted to develop more precisely quantified risk models for predicting cardiogenic stroke risk in non-valvular atrial fibrillation (NVAF) patients.

**Methods:** We conducted a case-control study, using data from hospitalized patients with AF who underwent transesophageal echocardiography at Shanghai Chest Hospital. A total of 233 high cardiogenic stroke risk patients with left atrial appendage thrombus (LAT) or left atrial spontaneous echo contrast (LA-SEC) and 233 controls matched for age, sex, AF type.

**Results:** AF history, LA diameter enlargement, larger left ventricular end diastolic diameter, lower ejection fraction, greater serum uric acid (SUA), and brain natriuretic peptide (BNP) levels showed association with high stroke risk. The multivariate logistic regression analysis revealed that AF duration, left atrial diameter (LAd), left ventricular ejection fraction (LVEF), SUA, and BNP were independent risk factors of the LAT/LA-SEC. We used LAd, LVEF, SUA, and BNP to construct a combined predictive model for high stroke risk in NVAF patients (the area under ROC curve: 0.784; sensitivity 66.1%; specificity 76.8%; 95% CI 0.744–0.825, *P* < 0.001).

**Conclusion:** Comprehensive evaluation of LAd, LVEF, SUA, and BNP may help stratify the cardiogenic stroke risk among non-valvular AF patients, guiding anticoagulation therapy.

## Introduction

Cardiogenic stroke is defined as the ischemic stroke caused by the shedding of a cardiogenic embolus and embolism corresponding to the cerebral artery. According to reports, it accounts for 14% of all ischemic strokes (1, 2). Atrial fibrillation (with or without other cardiovascular diseases) related stroke accounts for more than 79% of all cardiogenic stroke, which is the most important risk factor of cardiogenic stroke (3, 4). Compared with non-AF related stroke, AF related stroke has more severe symptoms, higher disability rate, higher mortality rate, and is easy to relapse; the mortality rate is twice as high as non-AF related stroke; the medical cost is 1.5 times as high as non-AF related stroke (5). Left atrial appendage thrombus (LAT) and left atrial spontaneous echo contrast (LA-SEC) caused by atrial fibrillation are high risk factors of cardiogenic stroke. The majority of AF is non-valvular AF. At present, esophageal ultrasound is still the gold standard for monitoring thrombus in left atrial appendage. Although there is evidence that standardized anticoagulation therapy can significantly improve the prognosis of patients with high risk of thromboembolic events, in fact, most patients with atrial fibrillation do not use anticoagulation therapy. Strategies for identifying patients at risk for thromboembolism are commonly based on the basis of the CHA2DS2 -VASc score (6), however study found biomarkers could further refine stroke risk differentiation among patients initially classified as low risk (7). Clinically, we also found patients with low CHA2DS2 -VASc score still have a risk of thromboembolic events, more valuable forecast indicators of biomarkers in patients with AF seems to be necessary. Therefore, we aimed to develop a more precisely quantified risk models for predicting cardiogenic stroke risk in non-valvular atrial fibrillation (NVAF) patients.

## Methods

### Study Population

The study population comprised 233 high cardiogenic stroke risk patients with LAT or LA-SEC and 233 controls matched for age, sex, AF type, between January 2017 and July 2019. AF was confirmed by a 12-lead surface electrocardiogram and Holter. Paroxysmal AF and non-paroxysmal AF were defined according to the published guideline. Stroke risk was then evaluated according to the Congestive Heart Failure, Hypertension, Age>75 Years, Diabetes Mellitus, Stroke, Vascular Disease, Age 65–74 Years, Sex Category (CHA2DS2 -VASc) score. All patients underwent echocardiography and TEE before catheter ablation, with written informed consent was obtained. LA thrombus was diagnosed by a well-circumscribed echogenic mass contrasted with the adjacent myocardium. LA-SEC was diagnosed by the presence of dynamic smog-like echoes in the left atrial cavity and left atrial appendage. The left atrial diameter (LAD) and left ventricular ejection fraction (LVEF) were measured by transthoracic two-dimensional echocardiography.

Data on the clinical baseline characteristics of all patients were collected from electronic medical records and analyzed. Patients were categorized into a thrombosis group and a normal group according to the TEE results. The Ethics Study Committee at Shanghai Chest Hospital approved the study protocols and agreed that informed consent was not necessary because of the observational nature of the study.

### Statistical Analysis

All analyses were performed with SPSS software version 25.0 (IBM Inc., NY, USA). All continuous data are presented as the mean ± SD deviation and were compared using Student *t*-test. Categorical variables were compared using Pearson's chi-square test or Fisher exact test whenever needed. The receiver operating characteristic (ROC) curve was constructed by plotting sensitivity vs. specificity used to discriminate the power of parameters in identifying the risk of stroke (LA/LAA thrombus, LAS-EC). Multivariable and univariable logistic regression was used to identify the risks of LAT or LA-SEC. All probability values were 2-sided and a *P* < 0.05 was considered statistically significant.

## Results

### Baseline Characteristics of the High Stroke Risk Group and Control Group

From January 1, 2017 to December 31, 2018, a total of 3,522 patients underwent TEE at the Shanghai Chest Hospital. After applying the exclusion criteria, 55 (1.56%) patients with non-valvular AF were LAT and 178 (5.05%) were LA-SEC. A case-control study was performed on 233 patients with LAT or LA-SEC and 233 age, sex, and AF-type matched control patients selected from a list of subjects who had undergone TEE. The baseline characteristics of patients in the high risk and control groups are summarized in Table 1.

**Table 1:** Baseline characteristics of the matched patient populations.

**Variables**	**High-risk (*n* = 233)**	**Control (*n* = 233)**	***P*-value**
Age, years	68.2 ± 7.9	68.2 ± 7.9	1.000
Male sex, *n* (%)	152 (65.2)	152 (65.2)	1.000
Per-AF, *n* (%)	205 (88.0)	205 (88.0)	1.000
AF history, years	3.8 ± 5.6	2.7 ± 3.5	0.010
Hypertension, *n* (%)	144 (61.8)	129 (55.4)	0.592
Diabetes mellitus, *n* (%)	38 (16.3)	30 (12.9)	0.295
Congestive heart failure, *n* (%)	34 (14.6)	19 (8.2)	< 0.001
Previous stroke/TIA, *n* (%)	40 (17.2)	39 (16.7)	1.000
Coronary artery disease, *n* (%)	29 (12.4)	30 (12.9)	0.890
Age ≥ 65	152 (65.2)	152 (65.2)	1.000
Age ≥ 75	44 (18.9)	44 (18.9)	1.000
CHA2DS2-VASc score, *n* (%)			0.080
0	11 (4.7)	25 (10.7)	0.023
1	40 (17.2)	45 (19.3)	0.632
≥ 2	182 (78.1)	163 (70.0)	0.057
Serum uric acid, μmol/L	406.4 ± 116.2	358.2 ± 78.2	< 0.001
Male	428.1 ± 121.3	376.0 ± 69.5	< 0.001
Female	365.7 ± 93.8	324.8 ± 83.1	0.004
Creatinine, μmol/L	79.6 ± 20.7	76.5 ± 16.6	0.147
Hematocrit, %	44.2 ± 10.0	43.0 ± 4.8	0.159
Platelets, 10 ^3^/μL	186.7 ± 63.2	188.9 ± 52.6	0.762
BNP, pg/mL	397.1 ± 403.8	188.0 ± 157.5	< 0.001
INR	1.31 ± 0.57	1.23 ± 0.54	0.358
INR ≥2.0, *n* (%)	27 (11.6)	19 (8.2)	< 0.001
Warfarin use, *n* (%)	67 (28.8)	49 (21.0)	0.277
NOAC, *n* (%)	13 (5.6)	17 (7.3)	0.572
Aspirin, *n* (%)	7 (3.0)	19 (8.2)	0.025
LAd, mm	47.5 ± 5.6	43.5 ± 5.1	< 0.001
LVDd, mm	50.1 ± 6.3	47.8 ± 4.2	< 0.001
LVEF, %	57.1 ± 9.5	62.2 ± 4.1	< 0.001
>Mild MR, *n* (%)	71 (30.5)	67 (28.8)	0.761

As shown in Table 1, the patients in high risk group had greater proportion of congestive heart failure, larger LA diameter, larger left ventricular end diastolic diameter, lower ejection fraction, greater SUA, and BNP than control group. The mean AF history (3.8 ± 5.6 vs. 2.7 ± 3.5 years, *P* = 0.01) was markedly longer in patients with LAT/LA-SEC. There were no statistically significant differences in hypertension, diabetes mellitus, previous stroke/TIA, coronary artery disease, CHA2DS2-VASc Score, and INR, more than moderate mitral regurgitation.

### Factors Predict High Stroke Risk and ROC Curve Analysis

Compared with the normal group, the high risk group had longer AF history, higher serum uric acid and BNP levels, LA enlargement, LVD enlargement, lower LVEF in high risk patients than in control group. All the above differences were statistically significant (*P* < 0.05). However, the CHA2DS2-VASc were similar between the two groups.

ROC curve analysis was conducted to evaluate the diagnostic value of statistically significant parameters in high risk patients (Table 2). The best cut-off value of SUA was ≥429.5 μmol/L (AUC 0.618, sensitivity 39.5%, specificity 83.7%, 95%CI 0.567–0.669, *P* < 0.001). The best cut-off value of BNP was ≥334.5 pg/mL (AUC 0.720, sensitivity 42.5%, specificity 88.8%, 95%CI 0.675–0.766, *P* < 0.001). The best cut-off value of LAd was ≥45.5 mm (AUC 0.705, sensitivity 61.8%, specificity 70.0%, 95%CI 0.659–0.752, *P* < 0.001). The best cut-off value of LVEF was ≤51.5% (AUC 0.677, sensitivity 73.4%, specificity 50.2%, 95%CI 0.629–0.725, *P* < 0.001).

**Table 2:** Receiver operating characteristic analysis of the risk factors.

	**Sensitivity (%)**	**Specificity (%)**	**AUC**	**95% CI**	***P*-value**
AF history	20.6	87.6	0.527	0.474–0.580	0.313
SUA	39.5	83.7	0.618	0.567–0.669	<0.001
BNP	42.5	88.8	0.720	0.675–0.766	<0.001
LAd	61.8	70.0	0.705	0.659–0.752	<0.001
LVDd	50.2	65.7	0.595	0.544–0.647	<0.001
LVEF	73.4	50.2	0.677	0.629–0.725	<0.001

### Multivariable Analysis for LAT or LA-SEC

Multiple candidate clinical predictors and echocardiography measurements was performed to identify the independent predictors for LAT/LA-SEC. Our results demonstrated that AF duration, LAd, LVEF, SUA, and BNP were significantly correlated with the presence of LAT/LA-SEC. Univariable and multivariable analysis showed that these parameters were found to be significantly predictive of high stroke risk in NVAF patients (Table 3).

**Table 3:** Univariable and multivariable logistic regression of cardiogenic stroke risk.

**Variable**	**Univariable**			**Multivariable**		
	**OR**	**95% CI**	***P*-value**	**OR**	**95% CI**	***P*-value**
SUA	1.005	1.003–1.007	<0.001	1.003	1–1.006	0.025
AF history	1.005	1.001–1.008	0.013	1.006	1.002–1.011	0.005
BNP	1.004	1.003–1.005	<0.001	1.002	1.001–1.003	0.006
LAd	1.160	1.113–1.209	<0.001	1.083	1.031–1.138	0.001
LVEDd	1.083	1.044–1.123	<0.001	0.969	0.916–1.025	0.275
LVEF	0.888	0.855–0.921	<0.001	0.9	0.858–0.944	<0.001

### Combined Predictive Model

**Figure 1:**
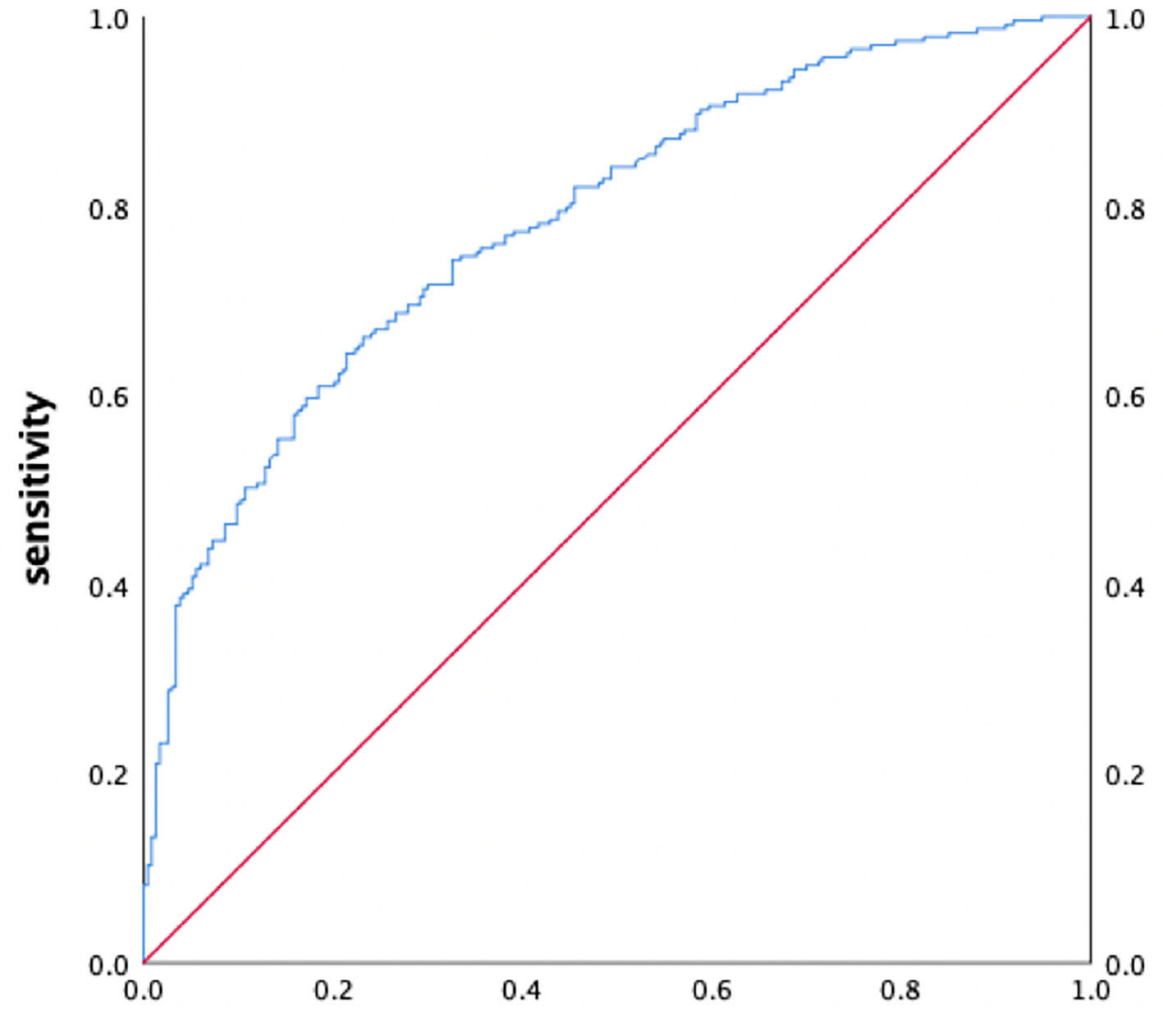
ROC curves analysis for predictive value of combined predictive model.

We used SUC, BNP, LAd, and LVEF as independent variables for further multivariate logistic regression. The results show that the combined predictive mode had an excellent discriminatory capacity in predicting high stroke risk (AUC 0.784; sensitivity 66.1%; specificity 76.8%; 95% CI 0.744–0.825, *P* < 0.001, Figure 1).

### Subgroup Analyses

Stratified analyses were performed to assess the predicted value of parameters in LAT and LA-SEC group. As shown in Table 4, the patients in LAT group had greater proportion of hypertension than LA-SEC group. There were no statistically significant differences in age, sex, AF type, AF history, diabetes mellitus, previous stroke/TIA, coronary artery disease, CHA2DS2-VASc Score, congestive heart failure, LAd, LVEDd, LVEF, creatinine, hematocrit, platelets, and use of anticoagulant or aspirin.

**Table 4:** Baseline characteristics of the LAT and LA-SEC patient populations.

**Variables**	**LAT (*n* = 55)**	**LAECS (*n* = 178)**	***P*-value**
Age, years	67.1 ± 8.9	67.2 ± 8.4	0.946
Male sex, *n* (%)	33 (60.0)	119 (66.9)	0.418
Per-AF, *n* (%)	52 (94.5)	151 (84.8)	0.067
AF history, years	3.8 ± 5.5	3.8 ± 5.6	0.973
Hypertension, *n* (%)	26 (47.3)	118 (66.3)	0.017
Diabetes mellitus, *n* (%)	6 (10.9)	32 (18.0)	0.296
Congestive heart failure, *n* (%)	12 (21.8)	22 (12.4)	0.124
Previous stroke/TIA, *n* (%)	2 (3.6)	38 (21.3)	0.002
Coronary artery disease, *n* (%)	4 (7.3)	25 (14.0)	0.244
Age ≥ 65	35 (63.6)	117 (65.7)	0.871
Age ≥ 75	11 (20.0)	33 (18.5)	0.844
CHA2DS2-VASc score, *n* (%)	2.6 ± 1.4	3.0 ± 1.7	0.080
0	4 (7.3)	7 (3.9)	0.293
1	9 (26.4)	36 (20.2)	0.696
≥ 2	42 (76.4)	135 (75.8)	1.000
Serum uric acid, μmol/L	427.7 ± 120.1	399.8 ± 114.5	0.120
Male	467.8 ± 134.0	417.1 ± 115.7	0.033
Female	367.5 ± 58.2	365.0 ± 104.4	0.913
Creatinine, μmol/L	79.6 ± 22.4	79.6 ± 20.2	0.996
Hematocrit, %	45.7 ± 17.4	43.8 ± 6.2	0.219
Platelets (10^3^/μL)	178.1 ± 68.5	189.3 ± 61.4	0.249
BNP	592.2 ± 624.9	336.8 ± 281.3	0.005
INR	1.25 ± 0.53	1.32 ± 0.58	0.452
INR ≥2.0, *n* (%)	6 (10.9)	21 (11.8)	1.000
Warfarin use, *n* (%)	14 (25.5)	53 (29.8)	0.611
NOAC, *n* (%)	1 (1.8)	12 (6.7)	0.310
Aspirin, *n* (%)	3 (5.5)	4 (2.2)	0.360
LAd, mm	48.8 ± 7.3	47.1 ± 5.0	0.122
LVDd, mm	51.5 ± 7.7	49.6 ± 5.7	0.095
LVEF, %	55.7 ± 12.4	57.5 ± 8.4	0.316
>Mild MR, *n* (%)	23 (41.8)	48 (27.0)	0.044

The SUA levels in LAT group were no statistically significant differences greater than in LA-SEC group. However, the mean male SUA level (467.8 ± 134.0 vs. 417.1 ± 115.7 μmol/L, *P* = 0.033) was significantly higher in patients with LAT than LA-SEC (Table 4).

**Figure 2:**
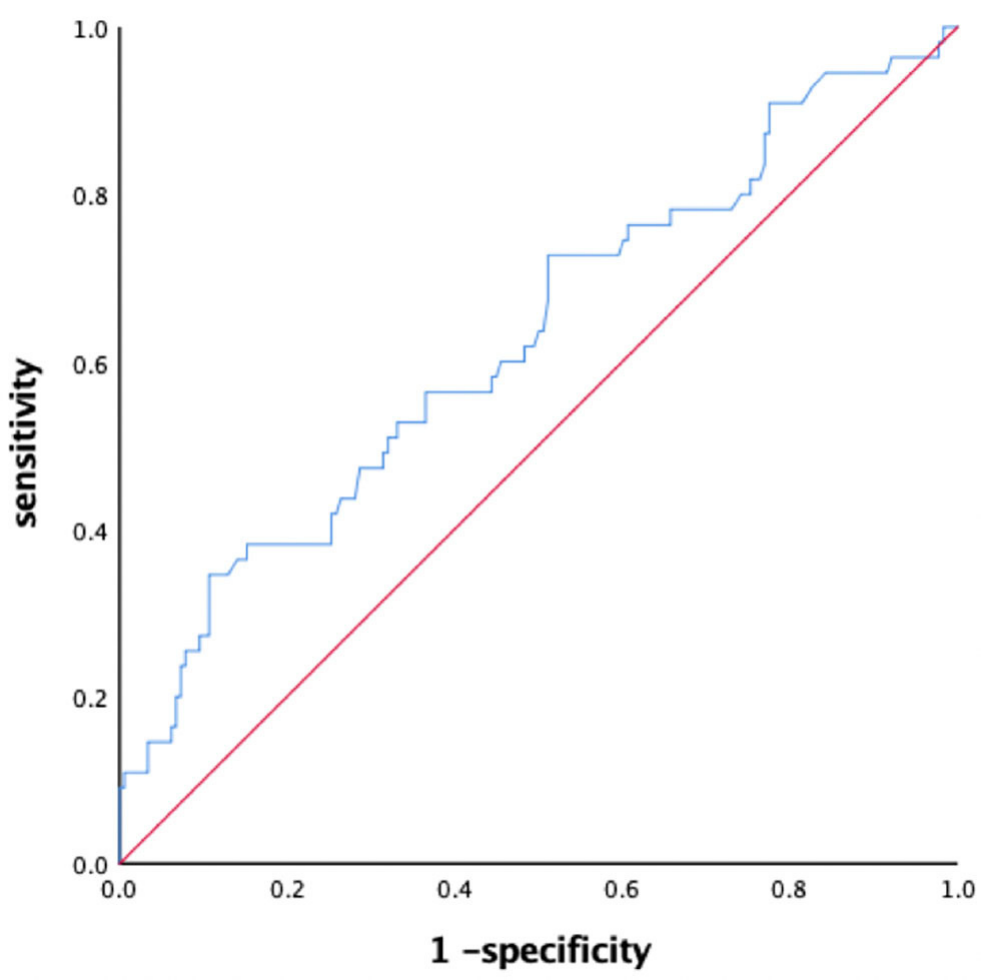
ROC curves analysis for predictive value of BNP level in LAT group.

The BNP level in LAT group were significantly greater than in LA-SEC group (592.2 ± 624.9 vs. 336.8 ± 281.3 pg/mL, *P* = 0.005). The corresponding AUC for BNP predicting LAT was 0.627 (95% CI: 0.539–0.715) and the best cut-off point for BNP predicting LAT was 627 pg/mL, the sensitivity and specificity were 34.5 and 89.3%, respectively (Figure 2).

## Discussion

### Main Findings

In this case-control study, we demonstrated a significant positive association between SUA, BNP, LAd, LVEF and high stroke risk in non-valvular atrial fibrillation patients. The main findings were as follows: (1) Patients in the LAT/LA-SEC group had significantly higher SUA, BNP levels, LAd and lower LVEF than the control group; (2) Increased SUA and BNP, LA enlargement, LVEF reduction were independent risk factors and combining these four factors above is stronger than using any one single factor for predicting high stroke risk in non-valvular AF patients; (3) BNP levels in LAT group were significantly higher than LA-SEC group, which can be a modest predictor of higher stroke risk in AF patients with LA-SEC.

SUA is the final product of purine metabolism catalyzed by xanthine oxidase, which plays an important role in the formation of free radical superoxide anion and oxidative stress, consequently resulting in calcium overload and decreasing sodium channels and aggravating cellular damage (8–10). These pathological processes promote electrical remodeling and structural remodeling of the left atrium, leading to an increase of its size and contribute to the occurrence and development of AF (11–15). High SUA level is an independent risk factor for stroke and cardiovascular death (16–19). Studies have shown that hyperuricemia is an important risk factor for stroke and may improve the clinical risk stratification of patients with atrial fibrillation (20). Although it is still unable to explain the mechanism of hyperuricemia and stroke. However, we found that patients with LAT and LASEC had higher SUA levels, which means hyperuricemia is associated with a high risk of cardiac stroke in patients with non-valvular atrial fibrillation. This may provide clues for screening high-risk groups and strengthening anticoagulation therapy.

BNP is a sensitive indicator reflecting the increase of cardiac pressure and volume load, and its level is related to the functional load of cardiac pump. When atrial fibrillation occurs, the left atrium cannot contract effectively, the damage of left ventricular diastolic function and the increase of left ventricular filling pressure can lead to left atrial blood stasis, presenting as SEC, and increase the risk of LAA thrombosis (21, 22). Studies have shown that BNP can predict the risk of atrial fibrillation (23), thromboembolism (22, 24–27), and general cardiovascular risk stratification in NVAF patients (28–30). Recent studies have suggested that BNP is not only a predictor of AF, but also an early predictor of cerebral embolism in patients with AF (31). Our study has demonstrated that BNP is associated with LAT and LA-SEC, and BNP levels in LAT patients are higher than those in SEC patients. BNP can predict the risk of cardiogenic stroke independently of CHADS 2 and CHA2DS 2-vasc scores, and a higher BNP value means a higher risk of stroke.

In our analysis, decreased LVEF was revealed to be a powerful and independent predictor of LAT/LA-SEC formation in AF patients, which means high cardiogenic stroke risk. Previous studies have suggested that incidence of LAT depending on LVEF, and severe LV systolic dysfunction (confirmed by echocardiography) was a strong predictor of stroke (32, 33).

Studies found that LA enlargement is association with LA-SEC and embolic events (34–36). Left atrial enlargement may lead to thrombotic stroke by promoting endothelial damage, atrial blood stasis, and thrombosis (37). Atrial cardiomyopathy caused by fibrosis of the left atrium can lead to atrial fibrillation over time. LA enlargement is the manifestation of the severity of atrial cardiomyopathy, and co-exist with AF (38).

Despite that CHA2DS2-VASc score is mostly used to predict the stroke risk in atrial fibrillation (39). However, we found that there was no relationship between the score and LAT/LA-SEC formation. In this study, we found that there was an independent correlation between SUA, BNP, LAd, LVEF and LAT/LA-SEC in AF patients, and subgroup analysis found that the BNP level in LAT group was higher than that in LA-SEC group, with significant statistical difference, which may provide clues for high BNP to increase the risk of cardiogenic stroke and risk in non-valvular AF patients.

## Clinical Implication

The main strength of our study was the generalization of the different features of the real-world non-valvular atrial fibrillation population in a matched cohort. Our present study found that a comprehensive evaluation of left atrial diameter, left ventricular ejection fraction, serum uric acid, and BNP may help stratify the cardiogenic stroke risk among non-valvular AF patients, which may help clinicians in the decision-guiding anticoagulation therapy.

## Limitations

The present study had several limitations. The number of patients is relatively insufficient to determine the actual prediction value of these parameters for LA-SEC. In addition, most of the study population met the criteria for catheter ablation of AF, so selection bias may limit the current statistical analysis, and the population in this study may not reflect all patients with non-valvular AF. Considering that this study is a retrospective study, further prospective clinical trials are necessary to verify the predictive value of these parameters on the risk of cardiogenic stroke caused by atrial fibrillation and the guiding significance of anticoagulation decision-making. The pathophysiological mechanism of LAT and LA-SEC has not been well-explored. The mechanism of combined predictive model for cardiogenic stroke risk in AF needs further study.

## Conclusion

This study found that the combined predictive model has a moderate predictive value for cardiogenic stroke risk among non-valvular AF patients, which will help us strengthen the screening of high-risk populations and strengthen anticoagulation therapy.

## Data Availability Statement

The original contributions presented in the study are included in the article/supplementary materials, further inquiries can be directed to the corresponding author/s.

## Author Contributions

All authors listed have made a substantial, direct and intellectual contribution to the work, and approved it for publication.

## Conflict of Interest

The authors declare that the research was conducted in the absence of any commercial or financial relationships that could be construed as a potential conflict of interest.
